# Everolimus initiated in the neonatal period for refractory seizures associated with tuberous sclerosis complex: long-term clinical outcome

**DOI:** 10.1016/j.ebr.2026.100887

**Published:** 2026-07-21

**Authors:** Yoong-A Suh, Seong Wan Kim, Seohui Choi, Moon Sung Park, Jang Hoon Lee, Soo In Jeong, Rita Yu, Young Bae Sohn

**Affiliations:** a Department of Pediatrics, Ajou University Hospital, Ajou University School of Medicine, Suwon, Republic of Korea; b Department of Medical Genetics, Ajou University Hospital, Ajou University School of Medicine, Suwon, Republic of Korea

**Keywords:** Tuberous sclerosis complex, mammalian target of rapamycin inhibitor, everolimus, neonatal seizures, cardiac rhabdomyoma

## Abstract

**Objective:**

Everolimus is an established therapy for tumor manifestations and refractory focal seizures in tuberous sclerosis complex (TSC) in patients aged ≥2 years. Evidence regarding its use during the neonatal period, particularly for seizure control, remains extremely limited.

**Methods:**

We describe the clinical course, neuroimaging findings, treatment response, and developmental outcome of a neonate with genetically confirmed TSC who received early everolimus therapy for refractory seizures.

**Results:**

A term female neonate developed multifocal drug-resistant seizures beginning on day 3 of life. Brain magnetic resonance imaging demonstrated multiple cortical and subcortical tubers, and electroencephalography revealed multifocal epileptiform discharges. Despite treatment with phenobarbital, midazolam, and vigabatrin, seizures remained uncontrolled. Everolimus was initiated on day 30 of life. Seizure activity resolved completely within one week and remained controlled for over one year. The treatment was well tolerated, with no serious adverse events. Early developmental assessment at 12 months showed cognitive, language, and motor scores within the lower range of normal.

**Conclusion:**

This case suggests that early initiation of everolimus may represent a feasible adjunctive treatment option for selected neonates with TSC-associated refractory seizures.

## Highlights

• Neonatal-onset refractory seizures in TSC successfully treated with early everolimus therapy.
• Rapid seizure resolution and sustained seizure freedom with acceptable safety profile.
• Early mTOR inhibition may modify epileptogenesis in high-risk neonatal TSC patients.

## 1 Introduction

Tuberous sclerosis complex (TSC) is a genetic multisystem disorder caused by pathogenic variants in *TSC1* or *TSC2*, resulting in dysregulation of the mammalian target of rapamycin (mTOR) signaling pathway [1], [2]. Epilepsy occurs in up to 90% of affected individuals and frequently presents during infancy, often with drug-resistant seizures that are strongly associated with adverse neurodevelopmental outcomes [3], [4], [5]. Early seizure onset, particularly during the neonatal period, is a major predictor of later cognitive impairment and autism spectrum disorder [3], [4], [6].

Everolimus, an mTOR inhibitor, has demonstrated efficacy for TSC-associated tumors and refractory focal seizures in children and adults. The phase III EXIST-3 trial established its role as adjunctive therapy for seizure control in patients aged ≥2 years [7], [8]. However, neonates were excluded from these trials, and evidence regarding the safety and efficacy of everolimus during the neonatal period remains scarce. Reported neonatal use has largely focused on the treatment of cardiac rhabdomyomas or other mass-related complications, rather than seizure control [9], [10], [11].

Here, we report a neonate with genetically confirmed TSC in whom everolimus was initiated during the neonatal period primarily for refractory seizure control. This case adds to the limited literature on early mTOR inhibition in neonatal epileptogenesis and highlights its potential therapeutic role in carefully selected cases.

## 2 Case presentation

**Fig. 1:**
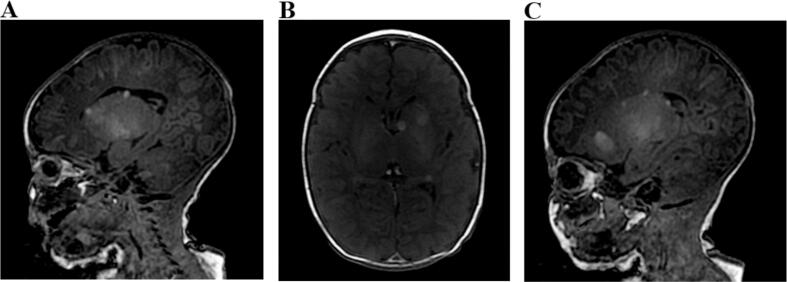
**Brain MRI findings consistent with tuberous sclerosis complex**Fig. 1: T1-weighted brain magnetic resonance images demonstrate multiple hyperintense nodules along the ventricular lining, consistent with subependymal hamartomas (A), including an approximately 6-mm lesion near the left foramen of Monro (B). A focal T1-hyperintense lesion in the subcortical white matter is also shown, compatible with a cortical/subcortical tuber (C).

A female infant was born at 39 + 5 weeks of gestation via cesarean section. Her birth weight was 3200 g, and Apgar scores were 8 and 9 at 1 and 5 min, respectively. She was the first child of non-consanguineous Korean parents with no family history of epilepsy or TSC. At 3 days of life, the infant developed intermittent focal limb jerking episodes, which increased in frequency over several days. She was admitted to the neonatal intensive care unit on day 9. Continuous electroencephalography confirmed multifocal epileptiform discharges and frequent electrographic seizures. Although overt clinical seizures were not observed daily, clinical seizures occurred approximately every other day, with 1–2 events per day when present. During 24-h electroencephalographic monitoring, frequent subclinical seizures lasting approximately 1 min occurred 4–5 times per hour, leading to midazolam coma therapy. Phenobarbital was initiated, followed by midazolam infusion and vigabatrin, but electrographic seizures persisted. Brain magnetic resonance imaging (MRI) revealed multiple T1-hyperintense nodules along the bilateral subependymal lining of the lateral ventricles, including an approximately 6-mm lesion near the left foramen of Monro, as well as multifocal cortical and subcortical tubers in the bilateral cerebral hemispheres, consistent with TSC (Fig. 1). Echocardiography demonstrated multiple cardiac rhabdomyomas associated with paroxysmal supraventricular tachycardia. Genetic testing using a next-generation sequencing epilepsy panel identified a heterozygous pathogenic nonsense variant in *TSC2* (c.4507C > T, p.Gln1503Ter), confirming the diagnosis of TSC. Given the refractory seizure course and multisystem involvement, off-label everolimus therapy was initiated on day 30 of life after obtaining parental consent and institutional approval. Phenobarbital was replaced with levetiracetam prior to treatment to minimize drug interactions. Everolimus was initiated at 0.25 mg once daily, corresponding to 0.065 mg/kg/day based on the body weight of 3.82 kg at treatment initiation. The dose was subsequently adjusted according to body weight and serum trough concentrations; at approximately 1 month after initiation, the dose was increased to 0.5 mg once daily, corresponding to 0.104 mg/kg/day based on a body weight of 4.8 kg. Serum everolimus trough concentrations were monitored serially and ranged from 1.4 to 15.5 ng/mL during follow-up (Table 1). After everolimus initiation, no further definite clinical seizures were observed. Electrographic seizure burden also decreased, with subclinical seizures occurring approximately once every 6 h on subsequent monitoring; vigabatrin was increased thereafter. The patient remained free of definite clinical seizures during hospitalization and was discharged at 2 months of age on everolimus, levetiracetam, and vigabatrin (Table 2).

**Table 1:** Everolimus dosing and safety monitoring during follow-up.⁎

Time point	BW	Everolimus dose	Dose mg/kg/day	Trough level, ng/mL	Safety monitoring
Initiation	3.82 kg	0.25 mg once daily	0.065	–	No baseline abnormality
5 days after	3.84 kg	0.25 mg once daily	0.065	1.4	No significant toxicity
1 months	4.8 kg	0.5 mg once daily	0.104	6.7	Mild intermittent diarrhea
1 years	10.5 kg	1 mg once daily	0.095	3.1	Mild intermittent diarrhea

BW, body weight.

⁎ Serial trough levels ranged from 1.4 to 15.5 ng/mL during follow-up. Safety monitoring included complete blood count, comprehensive metabolic panel, liver and renal function tests, and lipid profile. No clinically significant hematologic, hepatic, renal, or metabolic toxicity was observed, and treatment was not discontinued.

**Table 2:** Seizure burden, EEG findings, and antiseizure medication course.

Time point	Seizure burden	EEG findings	ASM
Before everolimus	Clinical seizures occurred approximately every other day, with 1–2 events per day when present	Multifocal epileptiform discharges; subclinical seizures lasting approximately 1 min occurred 4–5 times per hour	Phenobarbital, midazolam coma therapy, vigabatrin
After everolimus initiation	No further definite clinical seizures; subclinical seizures decreased to approximately once every 6 h	Decreased electrographic seizure burden	Everolimus added; levetiracetam and vigabatrin continued; vigabatrin increased
Discharge	No definite clinical seizures	–	Everolimus, levetiracetam, vigabatrin
Outpatient follow up	No definite recurrent clinical seizures	Abnormal background rhythms with occasional to frequent epileptiform discharges from the left frontal and right frontal-temporal areas	Everolimus and vigabatrin continued; levetiracetam discontinued; valproate and l-carnitine added

ASM, antiseizure medication; EEG, electroencephalography.

During outpatient follow-up, levetiracetam was discontinued, while everolimus and vigabatrin were continued. Valproate was subsequently added because of parental concern regarding recurrent sleep-related bilateral arm extension episodes, although these episodes were not clearly identified as epileptic seizures. l-carnitine was added concomitantly. Follow-up electroencephalography at approximately 1 year of age remained abnormal, with somewhat slow and disorganized background rhythms and occasional to frequent epileptiform discharges from the left frontal and right frontal-temporal regions, sometimes spreading to adjacent areas. Nevertheless, no definite recurrent clinical seizures were observed during follow-up.

Serial safety monitoring during everolimus treatment, including complete blood count, comprehensive metabolic panel, liver and renal function tests, and lipid profile, showed no clinically significant hematologic, hepatic, renal, or metabolic toxicity (Table 1). Mild intermittent diarrhea was observed but did not require treatment discontinuation.

Cardiac rhabdomyomas regressed progressively, and tachyarrhythmia resolved. At 12 months of age, developmental assessment using the Bayley Scales of Infant and Toddler Development, Third Edition (Bayley-III), showed cognitive, language, and motor composite scores of 90, 88, and 91, respectively, corresponding to the average to low-average range (Table 3). At the most recent follow-up at 14 months of age, the patient remained seizure-free with stable growth and development.

**Table 3:** Developmental assessment using the Bayley-III at 12 months of age.

Domain	Composite score	Classification
Cognition	90	Average
Language	88	Low average
Motor	91	Average

Bayley-III, Bayley Scales of Infant and Toddler Development, Third Edition.

## 3 Discussion

Epilepsy in TSC frequently begins in early infancy and is often highly drug resistant, particularly in patients with *TSC2* pathogenic variants [3]. Early seizure onset has been closely linked to unfavorable neurodevelopmental outcomes, underscoring the importance of rapid and effective seizure control. Although everolimus has emerged as a disease-modifying therapy targeting mTOR hyperactivation, clinical evidence supporting its antiseizure efficacy is largely derived from studies in older children and adults [7], [8]. This case is notable for the neonatal initiation of everolimus primarily for seizure control rather than for tumor-related indications. Despite failure of multiple antiseizure medications, seizures resolved rapidly after treatment initiation and remained controlled for more than one year. This temporal association suggests that mTOR inhibition may directly modulate epileptogenic mechanisms, rather than exerting antiseizure effects solely through tumor regression. Preclinical studies have demonstrated that mTOR hyperactivation contributes to aberrant neuronal connectivity and network hyperexcitability, and that mTOR inhibition can reduce epileptiform activity [12]. Clinical data from preventive strategies such as the EPISTOP trial further support the concept that early intervention in TSC-associated epilepsy may alter disease trajectory [6]. Although causality cannot be inferred from a single case, the favorable seizure control and early developmental outcomes observed here support the hypothesis that early mTOR pathway inhibition may influence epileptogenesis in high-risk neonates. Safety is a critical concern when considering everolimus therapy in neonates. Previous studies in infants younger than 12 months have reported generally acceptable tolerability of mTOR inhibitors, particularly when treatment is initiated with careful monitoring [5], [9]. Consistent with these findings, everolimus was well tolerated in this case under serial trough-level and laboratory monitoring, with no serious hematologic, hepatic, or metabolic adverse events.

In conclusion, this case adds to the limited evidence supporting early everolimus use for refractory neonatal seizures in TSC. While larger studies are required to define optimal dosing and long-term outcomes, early mTOR inhibition may represent a feasible therapeutic option in carefully selected neonates with severe, drug-resistant epilepsy.

## CRediT authorship contribution statement

**Yoong-A Suh:** Writing – review & editing, Writing – original draft, Conceptualization. **Seong Wan Kim:** Methodology, Data curation. **Seohui Choi:** Investigation, Data curation, Conceptualization. **Moon Sung Park:** Supervision, Methodology. **Jang Hoon Lee:** Writing – review & editing, Supervision. **Soo In Jeong:** Data curation, Conceptualization. **Rita Yu:** Data curation, Conceptualization. **Young Bae Sohn:** Data curation, Conceptualization.

## Ethical Statement

Ethical approval was obtained from the Institutional Review Board of Ajou University Hospital, Suwon, Republic of Korea (AJOUIRB-EX-2025-568). This study was conducted in accordance with the Declaration of Helsinki and its later amendments. Written informed consent was obtained from the patient's parents for publication of this case report and any accompanying images.

## Declaration of competing interest

The authors declare that they have no known competing financial interests or personal relationships that could have appeared to influence the work reported in this paper.
